# Under-five mortality according to maternal survival: a systematic review and meta-analysis

**DOI:** 10.2471/BLT.15.157149

**Published:** 2017-02-02

**Authors:** Lana Clara Chikhungu, Marie-Louise Newell, Nigel Rollins

**Affiliations:** a School of Languages and Area Studies, University of Portsmouth, Park Building, King Henry 1 Street, Portsmouth, PO1 2DZ, England.; b Academic Unit of Health and Development, University of Southampton, Southampton, England.; c Department of Maternal, Newborn, Child and Adolescent Health, World Health Organization, Geneva, Switzerland.

## Abstract

**Objective:**

To investigate, within so-called general populations, the relationship between maternal survival and mortality of children younger than five years.

**Methods:**

We conducted a systematic review of literature published between January 1990 and November 2016 that reported maternal vital status and the corresponding mortality of children younger than five years. Seven studies were included in a qualitative analysis and four in a random-effects meta-analysis. Summary estimates of the odds of dying by maternal survival were obtained and statistical heterogeneity estimated. Quality of the included studies and evidence was assessed using a Cochrane tool for assessing risk of bias and the Grading of Recommendations Assessment, Development and Evaluation criteria, respectively.

**Findings:**

Among children younger than five years, those whose mother had died were found to be 4.09 times (95% confidence interval, CI: 2.40–6.98) more likely to die than those with surviving mothers. Due to heterogeneity (I^2^: 83%), further pooled estimates were not possible. For children that were motherless as a result of maternal mortality, the increased odds of dying ranged from 1.40 (95% CI: 0.47–4.21) to 2.92 (95% CI: 1.21–7.04) among those aged between two and four years, 6.1 (95% CI: 2.27–16.77) to 33.78 (95% CI: 24.21–47.14) for those younger than one year and 4.39 (95% CI: 3.34–5.78) to 51.68 (95% CI: 20.26–131.80) for those younger than six months.

**Conclusion:**

The loss of a mother was associated with increased mortality among children, especially when maternal death occurred in the first year of the child’s life.

## Résumé

**Objectif:**

Étudier, au sein de la population dans son ensemble, la relation entre la survie maternelle et la mortalité des enfants âgés de moins de cinq ans.

**Méthodes:**

Nous avons effectué une revue systématique des écrits publiés entre janvier 1990 et novembre 2016 qui rendaient compte du statut vital de la mère et de la mortalité correspondante d'enfants âgés de moins de cinq ans. Sept études ont été incluses dans une analyse qualitative et quatre dans une méta-analyse à effets aléatoires. Nous avons obtenu des estimations récapitulatives des risques de décès en fonction de la survie de la mère et calculé l'hétérogénéité statistique. L'outil Cochrane, qui évalue le risque de biais, et les critères GRADE (Grading of Recommendations Assessment, Development and Evaluation) ont respectivement été utilisés pour mesurer la qualité des études et des données incluses.

**Résultats:**

Parmi les enfants âgés de moins de cinq ans, le risque de décès était 4,09 fois (intervalle de confiance, IC, à 95%: 2,40–6,98) plus élevé pour ceux dont la mère était décédée que pour ceux dont la mère était en vie. En raison de l'hétérogénéité (I^2^: 83%), il n'a pas été possible d'obtenir d'autres estimations groupées. Dans le cas des enfants orphelins de mère en raison de la mortalité maternelle, l'accroissement du risque de décès variait de 1,40 (IC à 95%: 0,47–4,21) à 2,92 (IC à 95%: 1,21–7,04) pour ceux âgés de deux à quatre ans, de 6,1 (IC à 95%: 2,27–16,77) à 33,78 (IC à 95%: 24,21–47,14) pour ceux âgés de moins d'un an et de 4,39 (IC à 95%: 3,34–5,78) à 51,68 (IC à 95%: 20,26–131,80) pour ceux âgés de moins de six mois.

**Conclusion:**

Le décès de la mère a été associé à une mortalité accrue chez les enfants, en particulier lorsqu'il intervenait au cours de la première année de vie de l'enfant.

## Resumen

**Objetivo:**

Investigar, dentro de la denominada población general, la relación entre la supervivencia materna y la mortalidad de niños menores de cinco años.

**Métodos:**

Se llevó a cabo una revisión sistemática de documentos publicados entre enero de 1990 y noviembre de 2016 que registraban el estado vital materno y la correspondiente mortalidad de niños menores de cinco años. Se incluyeron siete estudios en un análisis cualitativo y cuatro en un metaanálisis de efectos aleatorios. Se obtuvieron estimaciones de resumen de las probabilidades de fallecimiento por supervivencia materna y se estimó la diversidad estadística. Se evaluó la calidad de los estudios y las pruebas incluidos utilizando una herramienta de Cochrane para evaluar el riesgo de sesgo y los criterios de la Clasificación de la evaluación, desarrollo y valoración de las recomendaciones (GRADE), respectivamente.

**Resultados:**

Entre los niños menores de cinco años, aquellos cuyas madres murieron resultaron ser 4,09 veces (intervalo de confianza, IC, del 95%: 2,40–6,98) más propensos a morir que los niños cuyas madres sobrevivieron. Debido a la diversidad (I^2^: 83%), no fue posible realizar más estimaciones combinadas. Para los niños que quedaron sin madre debido a la mortalidad materna, el aumento de las probabilidades de muerte varió de un 1,40 (IC del 95%: 0,47-4,21) a un 2,92 (IC del 95%: 1,21–7,04) en aquellos niños de entre dos y cuatro años, de un 6,1 (IC del 95%: 2,27-16,77) a un 33,78 (IC del 95%: 24,21–47,14) en los menores de un año y de un 4,39 (IC del 95%: 3,34-5,78) a un 51,68 (IC del 95%: 20,26–131,80) en los menores de seis meses.

**Conclusión:**

La pérdida de la madre se asoció al aumento de la mortalidad infantil, sobre todo cuando el fallecimiento de la madre se produce en el primer año de vida del niño.

## ملخص

**الغرض:**

استقصاء العلاقة بين بقاء الأمهات على قيد الحياة ووفيات الأطفال من دون سن الخامسة ضمن ما يسمى بعموم السكان.

**الطريقة:**

أجرينا مراجعة منهجية للكتابات المنشورة في الفترة ما بين يناير/كانون الثاني 1990 ونوفمبر/تشرين الثاني 2016 التي تسجل الحالة الحيوية للأم والوفيات المقابلة من الأطفال دون سن الخامسة. وتم تضمين سبع دراسات في تحليل نوعي وأربعة في تحليل تلوي للآثار العشوائية. وقد تم الحصول على تقديرات الملخص لاحتمالات الوفاة بواسطة معدل بقاء الأمهات على قيد الحياة وقدر عدم التجانس الإحصائي. وتم تقييم جودة الدراسات المضمنة والأدلة باستخدام أداة من كوكرين لتقييم خطر الانحياز وتقييم التوصيات، ومعايير التطوير والتقييم، على التوالي.

**النتائج:**

وُجد أن الأطفال دون سن الخامسة ممن توفيت أمهاتهم من المحتمل وفاتهم بـ 4.09 مرة (بنسبة أرجحية مقدارها 95‏%: 2.40 – 6.98) أكثر ممن بقيت أمهاتهم على قيد الحياة. وفقًا لعنصر عدم التجانس (مربع معامل عدم التجانس I ^2 ^: 83‏%)، لم تكن التقديرات المُجمعة الإضافية محتملة. تراوحت احتمالات وفاة الأطفال بدون أمهات كنتيجة لوفاة الأم بنسبة من 1.40 (بنسبة أرجحية مقدارها 95‏‏%: 0.47 – 4.21) إلى 2.92 (بنسبة أرجحية مقدارها 95‏%: 1.21 – 7.04) ممن تراوح أعمارهم من سنتين إلى أربع سنوات، 6.1 (بنسبة أرجحية مقدارها 95‏%: 2.27–16.77) إلى 33.78 (بنسبة أرجحية مقدارها 95‏‏%: 24.21 – 47.14) للأطفال في عامهم الأول و4.39 (بنسبة أرجحية مقدارها 95‏‏%: 3.34 – 5.78) إلى 51.68 (بنسبة أرجحية مقدارها 95‏‏%: 20.26 – 131.80) للأطفال الذين تقل أعمارهم عن ستة أشهر.

**الاستنتاج:**

يرتبط فقدان الأم بزيادة معدلات الوفاة بين الأطفال، خصوصًا إذا توفيت الأم في السنة الأولى من عمر الطفل.

## 摘要

**目的:**

旨在在所谓的一般人群中调查孕产妇存活率与五岁以下儿童死亡率的关系。

**方法:**

我们对 1990 年 1 月至 2016 年 11 月期间发布的报告孕产妇死亡率状况及相应的五岁以下儿童死亡率的文献资料进行了系统性审查。 七项研究被纳入了定性分析，四项进行了随机荟萃分析。 对孕产妇存活率所致死亡概率进行了概括性估算并进行了统计学异质性估算。 分别使用考科蓝 (Cochrane) 偏倚风险评估工具与《证据推荐分级的评估、制订与评价》标准对所含研究及证据的质量进行了评估。

**结果:**

在五岁以下儿童当中，发现相较于母亲存活的儿童，母亲已去世儿童的死亡率高出 4.09 倍（95% 置信区间，CI： 2.40–6.98）。 由于异质性 (I^2^: 83%)，进一步汇集估算值不太可能。 对于因孕产妇死亡率造成的失母儿童，二至四岁儿童的死亡概率增加 1.40 （95% CI 0.47-4.21) 至 2.92 (95% CI: 1.21–7.04)，一岁以下儿童死亡概率增加 6.1 (95% CI: 2.27-16.77) 至 33.78 (95% CI： 24.21–47.14)，六个月以下儿童死亡概率增加 4.39 (95% CI： 3.34-5.78) 至 51.68 (95% CI： 20.26–131.80)。

**结论:**

母亲去世与儿童死亡率的增加有关，尤其是当孕产妇死亡发生在儿童出生后一年内。

## Резюме

**Цель:**

Изучить в рамках так называемых общих групп населения соотношение между материнской выживаемостью и смертностью детей в возрасте до пяти лет.

**Методы:**

Авторы провели систематический обзор литературы, опубликованной в период с января 1990 года по ноябрь 2016 года, в которой имеются данные о жизненном статусе матерей и смертности детей в возрасте до пяти лет. Семь исследований были включены в качественный анализ, и четыре — в метаанализ с использованием модели случайных эффектов. На основе материнской выживаемости были получены сводные оценки шансов смерти и была дана оценка статистической неоднородности. Качество включенных исследований и фактических данных оценивалось с помощью Кокрановского инструмента для оценки риска системной ошибки и критериев системы градации качества анализа, разработки и оценки рекомендаций (GRADE) соответственно.

**Результаты:**

Было обнаружено, что у детей в возрасте до пяти лет, чья мать умерла, в 4,09 раза (95%-й доверительный интервал, ДИ: 2,40–6,98) больше шансов умереть, чем у детей с выжившими матерями. По причине неоднородности (I^2^: 83%) дальнейшее проведение объединенной оценки было невозможным. Для детей, оставшихся без матери в результате материнской смертности, повышенные шансы смертности колебались от 1,40 (95%-й ДИ: 0,47–4,21) до 2,92 (95%-й ДИ: 1,21–7,04) среди детей в возрасте от двух до четырех лет, от 6,1 (95%-й ДИ: 2,27–16,77) до 33,78 (95%-й ДИ: 24,21–47,14) для детей младше одного года и от 4,39 (95%-й ДИ: 3,34–5,78) до 51,68 (95%-й ДИ: 20,26–131,80) для детей младше шести месяцев.

**Вывод:**

Утрата матери была сопряжена с повышенной смертностью среди детей, особенно когда материнская смерть наступала в течение первого года жизни ребенка.

Although child mortality has declined substantially since 1990, it remains a global health challenge, especially in resource-constrained settings.^1^ About 17 000 children die every day, the majority in sub-Saharan Africa and southern Asia.^1^ Socioeconomic status is central to a child’s survival because it affects several relevant maternal and environmental factors, nutritional status and risk of injury.^2^ The leading causes of mortality among those younger than five years are diarrhoea, low birth weight, prematurity, neonatal infections and respiratory infections.^1^

In 2013, 2% of global mortality among children younger than five years was attributed to human immunodeficiency virus (HIV) infection in the child.^3^ However, the successful roll-out of programmes for the prevention of mother-to-child transmission of HIV has led to fewer infants and children becoming infected with the virus each year.^4^ Attention is turning to the health and survival of the large numbers of children born to HIV-infected mothers who are themselves not infected with the virus. Compared with children born to HIV-uninfected mothers, such children still appear to show increased mortality.^5^^,^^6^ This may be associated with suboptimal infant feeding, which may be an adverse effect of attempts to prevent mother-to-child transmission and/or a consequence of maternal death or illness or other factors associated with the mother’s HIV infection.^5^^,^^7^^–^^12^ In one review, which examined the effect of parental death on child survival in all settings, maternal death was found to significantly increase the risk of death in childhood and, especially, death in infancy.^13^ However, this review did not stratify the children’s levels of survival by parental HIV status, even though this may be an important confounder.

To help elucidate the association between maternal death and the risk of mortality in HIV-uninfected children born to HIV-infected women, we conducted this systematic review. Our aim was to compare, in so-called general populations, the risk of death among children who were motherless as a result of maternal mortality to that of other children. We failed to identify any prior systematic reviews on this topic. Our hope was that the results of our review would help to determine whether the increased mortality reported among children following the deaths of their HIV-infected mothers can be entirely attributed to HIV or might be at least partially attributable to loss of maternal care.

## Methods

We conducted a systematic review based on a Population, Intervention, Comparison and Outcome framework.^14^ Our aim was to investigate whether, in populations not affected by HIV, maternal death increased mortality among children younger than five years. Our review included both experimental and observational studies – i.e. cohort, cross-sectional, longitudinal and randomized controlled studies – in which the study participants were mothers and children younger than five years from so-called general populations. Death and illness of the mother were considered as separate exposures. The primary outcome was death in children younger than five years. The time interval between the maternal death and the corresponding child death was a secondary outcome measure. We excluded studies that specifically recruited mothers living with HIV, studies that did not provide estimates of mortality for children younger than five years and studies that focused on pregnancy-related maternal deaths or HIV-infected children.

We searched the CINAHL, Delphis, Medline, PubMed and Web of Knowledge databases and the website of the International Union for the Scientific Study of Population’s 2013 conference^15^ for potentially relevant articles that were published, in English, between 1 January 1990 and 30 November 2016 (Box 1). The reference lists of articles that were considered relevant – by both authors involved in this stage of the study (LC and MLN) – were also searched. Four of the seven articles included in the qualitative analysis were also included in the meta-analysis.

Box 1 **Database search strategy to find studies on mortality among children younger than five years**

Titles and abstracts of articles published in English were searched for the following terms: ((((((((((“maternal mortality”[All Fields] OR “maternal death”[All Fields]) OR “maternal survival”[All Fields]) OR ((“mothers”[MeSH Terms] OR “mothers”[All Fields] OR “maternal”[All Fields]) AND vital[All Fields] AND status[All Fields])) OR “mother* death”[All Fields]) OR “mother* survival”[All Fields]) OR “parental death”[All Fields]) OR “parental survival”[All Fields]) OR “parent's death”[All Fields]) OR (parent's[All Fields] AND (“mortality”[Subheading] OR “mortality”[All Fields] OR “survival”[All Fields] OR “survival”[MeSH Terms]))) AND (((“child mortality”[All Fields] OR “under-five mortality”[All Fields]) OR “child death”[All Fields]) OR “infant death”[All Fields]) OR “infant survival”[All Fields]) OR “child survival”[All Fields] NOT (“hiv”[MeSH Terms] OR “hiv”[All Fields]) AND (“1990/01/01”[PDAT]: “2016/11/30”[PDAT])

To evaluate the quality of the included studies, we used a Cochrane tool for assessing risk of bias.^16^ In the development of the review and in the preparation of the final manuscript, we were guided by the AMSTAR tool.^17^

### Statistical analysis

Data on maternal and child deaths in publications were captured and summarized using version 5.3 of the Review Manager software package.^18^ For each of the studies included in the analysis, odds ratios (OR) and standard errors were either extracted from the published reports or computed from the raw data. Studies with a similar measure of effect, study design and children’s age groups were pooled. Random-effects analysis models^19^ were used to obtain summary estimates of the children’s odds of dying and the corresponding 95% confidence intervals (CI). These results were used to produce a forest plot. Heterogeneity was assessed using the *I^2^* statistic and values of this statistic between 50% and 90% were considered indicative of considerable heterogeneity.^20^ We conducted a sensitivity analysis by assessing each study’s contribution to the heterogeneity score and made a decision to include or exclude a study dependent on its contribution to the heterogeneity score and its risk of bias. A narrative qualitative analysis was adopted for the relevant studies that could not be summarized through a meta-analysis. The quality of the body of evidence was assessed using the Grading of Recommendations Assessment, Development and Evaluation criteria and summarized in evidence profiles.^21^ The small number of studies included in our review meant that it was not appropriate to use funnel plots to explore the potential for publication bias.^22^ However, since the deaths of mothers and children occur naturally, we considered the potential for such bias to be small.

### Ethics statement

The study was exempted from ethics review as all the data used had already been published.

## Results

**Fig. 1:**
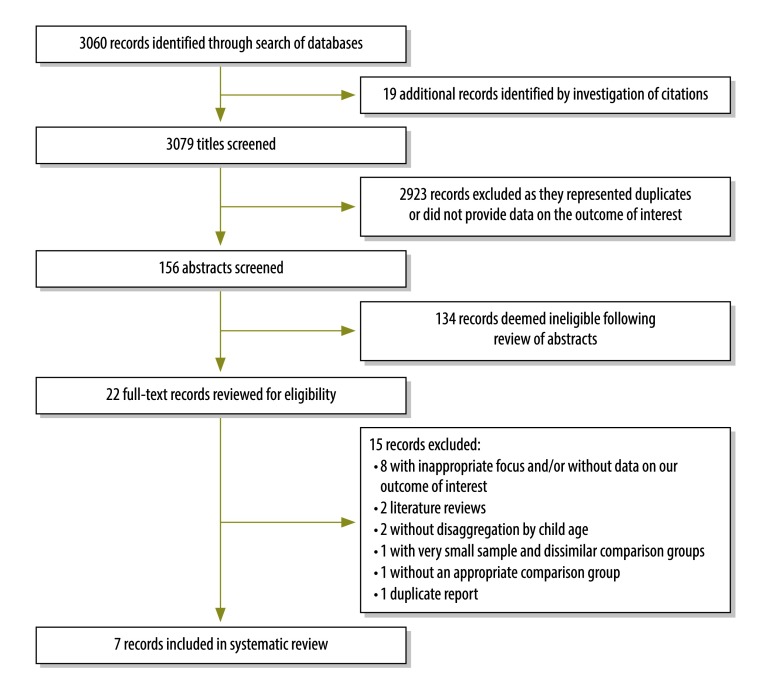
Flowchart showing the selection of studies on mortality among children younger than five years

The search of databases identified 3060 potentially relevant articles and an additional 19 such articles were identified from secondary bibliographical searches (Fig. 1). In the review of abstracts, the same 22 articles were deemed potentially eligible for inclusion in the systematic review by each of the two authors involved. Subsequently, 15 of these 22 articles were excluded (Fig. 1). We considered all seven studies included in the review to be observational: four reported data from demographic surveillance sites,^23^^–^^26^ two reported population or civil registration data^27^^,^^28^ and one, which provided data from a randomized control study,^29^ was not randomized for our outcome of interest (Table 1). Three of the seven studies were considered ineligible for the meta-analysis (Fig. 1).^27^^–^^29^ Data extracted from the seven studies included in the review and information on background maternal and child mortality rates obtained from the World Health Organization’s (WHO’s) Global Health Observatory website^30^ are available from the corresponding author.

**Table 1:** Summary of the seven studies included in the systematic review of under-five mortality according to maternal survival status

Publication	Study				Included in meta-analysis
	Design	Period	Country	Subjects	
Sear et al.^27^	Birth and death registers	1950–1974	Gambia	2294 children aged 0–5 years	No
Katz et al.^29^	Randomized controlled trial^a^	1994–1997	Nepal	15 469 infants aged 4–24 weeks	No
Reher and González-Quiñones^28^	Birth and death registers	1870–1950	Spain	20 895 children aged 0–5 years	No
Becher et al.^26^	Cohort	1992–1999	Burkina Faso	10 122 children aged 0–5 years	Yes
Masmas et al.^23^	Cohort	1990–1997	Guinea-Bissau	1127 children aged 0–5 years	Yes
Ronsmans et al.^24^	Cohort	1982–2005	Bangladesh	136 368 children aged 0–5 years	Yes
Clark et al.^25^	Cohort	1994–2008	South Africa	4584 children aged 0–5 years	Yes

^a^ Participants were randomized to receive or not to receive a nutritional supplement. They were not randomized according to maternal survival.

The quality of the relevant evidence provided by all seven studies included in our systematic review was initially categorized as low because of the studies’ observational design. The quality of evidence provided by two of the studies^27^^,^^28^ was subsequently downgraded to very low because data from the birth and death registers used may have missed children that were not registered or those that died before they could be registered (available from the corresponding author). All seven studies were considered to have a medium risk of bias because they could not be considered comparable to a well-performed randomized trial (Table 2). No studies could be assessed in terms of the selection of participants, departures from intended interventions or missing data – mainly because death of mother could not be treated as if it were an intervention.

**Table 2:** Assessment of risk of bias in studies on mortality among children younger than five years

Study	Confounding	Selection of participants into the study^a^	Measurement of interventions	Departures from intended interventions^a^	Missing data^a^	Measurement outcomes	Selection of the reported results	Overall
Sear et al.^27^	Medium risk	N/A	Low risk	N/A	N/A	Low risk	Low risk	Medium risk
Katz et al.^29^	Medium risk	N/A	Low risk	N/A	N/A	Low risk	Low risk	Medium risk
Reher and González-Quiñones^28^	Medium risk	N/A	Low risk	N/A	N/A	Low risk	Low risk	Medium risk
Becher et al.^26^	Medium risk	N/A	Low risk	N/A	N/A	Low risk	Low risk	Medium risk
Masmas et al.^23^	Medium risk	N/A	Low risk	N/A	N/A	Low risk	Low risk	Medium risk
Ronsmans et al.^24^	Medium risk	N/A	Low risk	N/A	N/A	Low risk	Low risk	Medium risk
Clark et al.^25^	Medium risk	N/A	Low risk	N/A	N/A	Low risk	Low risk	Medium risk

N/A: not applicable.

^a^ The risk of bias assessment with respect to “Selection of participants,” “Departures from intended interventions” and “Missing data” were not relevant to this study due to death of mother not being a typical intervention undertaken by nonrandomized studies.

Note: Risk of bias was assessed using the ROBINS–I tool.^16^

### Mortality of children

#### According to maternal survival

**Fig. 2:**
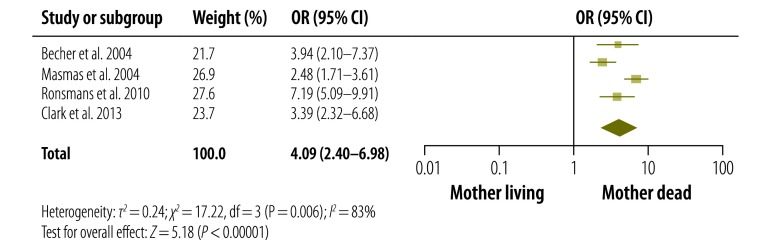
Odds of dying for children younger than five years whose mother had died – compared with other children in the same age group

CI: confidence interval; df: degrees of freedom; OR: odds ratio.

Note: This forest plot summarizes the main results of a random-effects meta-analysis of data from four studies.^23^^–^^26^

Due to differences in the ages of the children studied, measures of effect and study design, only four studies could be included in the pooled analysis of the odds of dying for children younger than five years.^23^^–^^26^ The results of the meta-analysis are presented in Fig. 2.

For children whose mothers had died, the overall risk of dying before they reached an age of five years was more than four times higher than the corresponding risk for children whose mothers survived (OR: 4.09; 95% CI: 2.40–6.98). However, there was considerable statistical heterogeneity (*I^2^*: 83%) between the four studies included in the meta-analysis. In our sensitivity analysis, we found that removal of one study^24^ from the meta-analysis reduced heterogeneity considerably (*I^2^* = 26%) and reduced the corresponding OR – for the death of motherless children before an age of five years, compared with that of other children – to 3.16 (95% CI: 2.27–4.38). However, since there was no difference in the risk of bias across the four studies, we decided to leave data from all four in the final meta-analysis.

It was not possible to undertake a meta-analysis according to the age group of the children involved – e.g. of children younger than six months, younger than one year, one to two years and two to four years – because of the small number and the differences in study design of the eligible studies. However, individual study estimates of the age-related risks of mortality are presented in Table 3.

**Table 3:** Age-related odds of dying for young children whose mother had died – compared with other children in the same age groups

Child’s age	Publication	Country	OR (95% CI)
< 6 months	Katz et al.^29^	Nepal	51.68 (20.26–131.80)
< 6 months	Reher and González-Quiñones^28^	Spain	4.39 (3.34–5.78)
< 6 months	Ronsmans et al.^24^	Bangladesh	36.23 (24.97–52.58)
6–11 months	Reher and González-Quiñones^28^	Spain	2.27 (1.56–3.29)
< 1 year	Sear et al.^27^	Gambia	6.17 (2.27–16.77)
< 1 year	Ronsmans et al.^24^	Bangladesh	33.78 (24.21–47.14)
1–2 years	Sear et al.^27^	Gambia	5.21 (1.58–17.21)
2–4 years	Sear et al.^27^	Gambia	1.40 (0.47,4.21)
2–4 years	Becher et al.^26^	Burkina Faso	2.92 (1.21,7.04)

CI: confidence interval; OR: odds ratio.

#### According to timing of maternal death

The association between risk of child death and timing of maternal death was addressed in only two of the seven studies.^23^^,^^25^ Narrative synthesis of these two studies indicated that the risk of the child dying was increased nearer to the timing of the mother’s death.

Data from a rural South African demographic surveillance site, collected between 1994 and 2008, have been analysed, with the OR for child mortality adjusted for potentially confounding characteristics of the children and mothers. The results indicated that the probability of a child dying started to increase six to eleven months before the child’s mother’s death and increased markedly during the two months immediately before the month of the mother’s death (adjusted odds ratio, aOR: 7.1; 95% CI: 3.7–12.7).^25^ The odds of child death were highest in the month of the mother’s death (aOR: 12.6; 95% CI: 6.2–25.3).^25^

Similar findings were reported from Guinea-Bissau.^23^ In urban Guinea-Bissau, the mortality rate ratio for children whose mothers had died – compared with children whose mothers were still alive – was estimated to be 3.09 (95% CI: 1.27–7.49) over the period from the death of the mother to five months later. If the child was still alive six months after the maternal death, then the child’s subsequent mortality risks were not significantly different from those of children whose mothers remained alive. In rural areas of Guinea-Bissau, the mortality rate ratios over the period from the death of the mother to five months later and from six months after the maternal death were 5.93 (95% CI: 3.44–10.26) and 2.56 (95% CI: 1.29–5.09), respectively.^23^

## Discussion

We found only seven papers suitable for inclusion in our systematic review and only four of these were considered eligible for meta-analysis. All of the latter demonstrated that children younger than five years were at an increased risk of death after their mothers died.^23^^–^^26^ Although this pattern was also reflected in the narrative analysis of age-related child mortality, the corresponding pooled estimates could not be made because of the differences in study design.^24^^,^^26^^–^^29^ Mortality risks were especially increased for motherless infants younger than six months. The increased likelihood of a child dying, as an apparent consequence of maternal death, was statistically significant among children aged between one and two years but not for children aged between two and four years. Results from two studies strongly indicated that children were more likely to die around the time of a maternal death^23^^,^^25^ than six or more months after the mother’s death.

The increased risk of death we observed for children left motherless in the first six months of their lives may reflect the particular vulnerability of infants^31^ and explain why maternal sickness in the first six months postpartum may have such serious consequences.^32^ Mothers who are ill may not be able to provide adequate care for their children – e.g. they may be unable to provide optimal breastfeeding – and this may jeopardize the children’s health and nutritional status.^32^^–^^35^ Premature weaning, which is associated with higher mortality rates, especially for younger infants, may occur.^7^^,^^23^^,^^33^^,^^36^ In settings where infant survival is highly dependent on continued breastfeeding, the loss of a father may not increase the risk of a child dying to the same extent as the loss of the mother.^37^^,^^38^ Even though adoption and remarriage may protect children left motherless as a result of maternal mortality, the quality of care received by such children may be lower than the care they would have otherwise received from their biological mothers.^31^^,^^39^^–^^41^

Although access restrictions prevented us from searching Embase, we believe that our careful search of the CINAHL, Delphis, PubMed and Web of Knowledge databases, our follow-up of the reference lists from potentially relevant articles and our scrutiny of abstracts from a large, international and relevant conference means that we are unlikely to have missed any important publications.

The considerable variation in child mortality risks observed across the studies we reviewed could reflect differences in background infant and child mortality rates related to demographic, environmental and/or socioeconomic factors and/or differences in the health and social support systems available. The quality of data may also have been variable. Civil registration data in developing countries are often incomplete and may not capture deaths as well as data from demographic surveillance sites. Similarly, historical data – e.g. data from church death registers in developed countries – are likely to have missed pre-baptism deaths. Differences in the methodological approaches followed may also account for some differences in the estimated risks. For example, Sear et al.^27^ used multilevel modelling to obtain the median odds of dying within Gambian households while other studies modelled the odds of dying at the individual level. The quality of the data in our included studies also ranged from low to very low. Despite the variations between the studies we investigated, the estimates of increased risk following maternal death were consistent and all in the same direction – even if, in some settings, the small sample sizes may have prevented statistical significance being reached.

In conclusion, between birth and an age of five years, children in so-called general populations who are left motherless as a result of maternal mortality are more likely to die than other children. The vulnerability of these children is highest in infancy, especially in the first six months of life. If the care and nutrition of motherless young children can be targeted by their communities and national health systems, the survival of such children could be improved.
